# Interleukin (IL)-21 in Inflammation and Immunity During Parasitic Diseases

**DOI:** 10.3389/fcimb.2019.00401

**Published:** 2019-12-04

**Authors:** Shahram Solaymani-Mohammadi, Lars Eckmann, Steven M. Singer

**Affiliations:** ^1^Center for Global Infectious Disease Research, Seattle Children's Research Institute, Seattle, WA, United States; ^2^Department of Medicine, University of California, San Diego, La Jolla, CA, United States; ^3^Department of Biology, Georgetown University, Washington, DC, United States

**Keywords:** interleukin (IL)-21, parasite, inflammation, immunity, cytokine, signaling

## Abstract

Parasitic diseases cause significant morbidity and mortality in the developing and underdeveloped countries. No efficacious vaccines are available against most parasitic diseases and there is a critical need for developing novel vaccine strategies for care. IL-21 is a pleiotropic cytokine whose functions in protection and immunopathology during parasitic diseases have been explored in limited ways. IL-21 and its cognate receptor, IL-21R, are highly expressed in parasitized organs of infected humans as well in murine models of the human parasitic diseases. Prior studies have indicated the ability of the IL-21/IL-21R signaling axis to regulate the effector functions (e.g., cytokine production) of T cell subsets by enhancing the expression of T-bet and STAT4 in human T cells, resulting in an augmented production of IFN-γ. Mice deficient for either IL-21 (*Il21*^−/−^) or IL-21R (*Il21r*^−/−^) showed significantly reduced inflammatory responses following parasitic infections as compared with their WT counterparts. Targeting the IL-21/IL-21R signaling axis may provide a novel approach for the development of new therapeutic agents for the prevention of parasite-induced immunopathology and tissue destruction.

## Introduction

Parasitic diseases pose a major public threat to the world's population, causing immense morbidity and mortality, especially in underdeveloped and developing countries (Mackey et al., 2014; Torgerson et al., 2014, 2015). Parasites colonize and replicate in a wide range of human tissues and clinical symptoms can range from a completely asymptomatic profile to severe disease profiles that can be either transient or chronic (Solaymani-Mohammadi and Singer, 2010). In immunocompetent individuals, most parasitic infections are generally considered self-limited, indicating the development and the maintenance of protective immune mechanisms against invading parasites (Okhuysen, 2001; Meamar et al., 2006; Saporito et al., 2013; Lanocha-Arendarczyk et al., 2018). However, multiple studies have suggested that host immune responses elicited during parasitic infections can mediate immunopathology and are responsible for many of the symptoms commonly observed during parasitic diseases (Phillips and Fox, 1984; Pesce et al., 2006; Babaei et al., 2016; Ivanova et al., 2019; Weaver et al., 2019). These immunopathological changes may include parasite-induced morphological, functional, physiological, and structural alterations in parasitized tissues/cells, rendering infected individuals susceptible to organ dysfunction as well as the development of severe forms of clinical disease (Solaymani-Mohammadi and Singer, 2013; Taniguchi et al., 2015; Gorosito Serran et al., 2017; Ma'ayeh et al., 2018).

Increasing evidence suggests that immune responses meant to eliminate or restrict the invading tissue-residing parasites can drive organ fibrosis and the subsequent organ dysfunction (Pesce et al., 2006; Nair et al., 2011; Adams et al., 2014; Sripa et al., 2018; Ferreira et al., 2019). For example, continuous and chronic infection episodes of the liver in humans infected with the blood fluke, *Schistosoma mansoni*, or cystic hydatidosis caused by the larval stage of *Echinococcus granulosus* can eventually lead to severe forms of liver fibrosis (Zhang C. et al., 2016; Labsi et al., 2018; Yong et al., 2019). It has been suggested that T_H1_ and T_H2_ immune responses have opposing functions during human schistosomiasis; T_H2_ polarization, mainly in response to *Schistosoma* egg antigens, promotes liver fibrosis during human schistosomiasis, whereas a T_H1_-biased immune response inhibits the development of liver fibrosis during schistosomiasis (Mentink-Kane and Wynn, 2004; Wynn, 2004; Wynn et al., 2004). Increased levels of T_H1_-associated cytokines, such as TNF-α and IFN-γ, are observed in the blood and tissues in different clinical presentations of malaria, suggesting pivotal roles played by these cytokines in protection against malaria in both rodent and human malaria (Hernandez-Valladares et al., 2006; Poovassery et al., 2009; Korner et al., 2010; Fauconnier et al., 2012). However, it is well-established that enhanced levels of TNF-α are associated with a greater risk of the development of severe malaria in humans and mice deficient for this cytokine were resistant to the development of cerebral malaria (CM) following *Plasmodium berghei* infection (Rudin et al., 1997; Perera et al., 2013; Dunst et al., 2017). Further analyses have indicated that proinflammatory cytokines, such as TNF-α, can regulate the expression of chemokine/chemokine receptors and other adhesion molecules by endothelial cells of the brain and they can enhance the trafficking and the homing of inflammatory leukocytes to the brain, leading to an exacerbated disease profile (Pober and Sessa, 2007; Miu et al., 2008).

Due to a variety of factors, including elaborate mechanisms of antigenic variation and immune evasion used by many parasites, there have been no effective vaccines approved for humans against parasitic diseases. This lack of efficacious vaccines for most parasitic infections in humans coupled with the increasing emergence of drug-resistant parasite strains requires the continuous development of new therapeutic approaches. A comprehensive understanding of the host-parasite interactions as well as the cytokine crosstalk following parasitic infections, including identification of the mechanisms by which protective and deleterious immune responses are regulated, can help design novel strategies for fighting parasitic infections and reducing the associated morbidity and mortality.

## IL-21/IL-21R Signaling Pathway

IL-21 is a member of the common gamma chain (γc) family of cytokines and is expressed by multiple immune cell types (Spolski and Leonard, 2008), with activated CD4^+^ T cells, including T follicular helper (T_FH_) cells and natural killer (NK) T cells, being the major sources of this cytokine (Spolski and Leonard, 2010a; Crotty, 2011; Linterman et al., 2011). The induction of IL-21 in activated CD4^+^ T cells is mediated by c-Maf *in vitro* (Hiramatsu et al., 2010; Kroenke et al., 2012) and *in vivo* (Bauquet et al., 2009), whereas the expression of c-Maf in CD4^+^ T cells is regulated by IL-6 (Hiramatsu et al., 2010) or IL-27 (Pot et al., 2009).

The biological functions of IL-21 are mediated by binding to its corresponding receptor, IL-21R. IL-21R is expressed by a wide range of immune cells, including T and B cells, NK cells, DCs and macrophages as well as non-immune cells, including epithelial cells and keratinocytes (Distler et al., 2005; Caruso et al., 2007; Crotty, 2011). The ubiquitous expression of the IL-21R may explain the broad biological functions of IL-21 on the cells of hemopoietic and non-hemopoietic origins. The IL-21/IL-21R signaling activates the Janus kinase (JAK1/3)-signal transducer and activator of transcription (STAT) signaling pathway (Spolski and Leonard, 2008). Accordingly, the phosphorylated STAT proteins are dimerized and translocated into the nucleus, where they bind to interferon (IFN)-γ-activated sequence (GAS) elements and initiate a gene transcription profile (i.e., *Gzma, Gzmb, Il10, Eomes, Rorgt*) (Spolski and Leonard, 2014). IL-21 exerts its regulatory functions on the target cells predominantly via the activation of STAT3 but it also recruits STAT1 and STAT5 (Spolski and Leonard, 2008; Wan et al., 2013). The cascade of signaling events downstream of the IL-21-induced activation of STAT3 is well-characterized, however, what is still unclear is how the activation of STAT1 by IL-21 regulates the expression of the downstream IL-21 target genes (Wan et al., 2013). Interestingly, the IL-21-induced activation of STAT1 leads to the augmented expression of *Tbx21* and *Ifng* genes (Wan et al., 2015).

T_FH_ cells are considered one of the major sources of the IL-21 production (Spolski and Leonard, 2008, 2010a,b). These cells are a specialized subset of CD4^+^ T cells that can promote T cell-dependent humoral immune responses (Zotos et al., 2010; Rankin et al., 2011; Achour et al., 2017). Multiple signaling pathways contribute to the differentiation and the development of T_FH_ cells, among which IL-6 and the inducible T-cell costimulator (ICOS) ligand or ICOSL (CD275) have been shown to be important in the early differentiation of these cells in the mouse (Vinuesa et al., 2016). T_FH_ cells are identified by several surface markers, including CXCR5, ICOS, PD-1 and, in addition to IL-21, these cells canonically secret C-X-C motif chemokine 13 (CXCL13) and IL-4 (Crotty, 2011; Liang et al., 2011; Linterman et al., 2011; Kroenke et al., 2012). CXCL13 is expressed predominantly by non-T_FH_ cell sources (i.e., stromal cells) in mice, whereas T_FH_ cells are the major source of this molecule in humans (Pitzalis et al., 2014; Crotty, 2019). The transcription factor, B-cell lymphoma 6 (Bcl6) is required for the differentiation of T_FH_ cells (Nurieva et al., 2009; Hollister et al., 2013), however, other transcription factors have been shown to regulate the differentiation of T_FH_ cells via the induction of Bcl6 (Reviewed in Crotty, 2019).

Germinal centers (GCs) are specialized locations in the secondary lymphoid tissues within which B cells proliferation, antibody somatic hypermutation and the affinity maturation (i.e., generation and selection of B cells with high affinity antibody secretion) occur (Zotos et al., 2010; Mesin et al., 2016). Severe impairment in B cells response to antigen of protein origin, a significant reduction in plasma cell formation in both the spleen and the bone marrow were observed in mice lacking IL-21 or its cognate receptor, IL-21R, *Il21*^−/−^, and *Il21r*^−/−^mice, respectively (Zotos et al., 2010). It has been proposed that the IL-21/IL-21R signaling regulates the Bcl6 expression by B cells and the T_FH_-derived IL-21 promotes the transition of the peri-follicular pre-GC B cell to the intrafollicular phase (Linterman et al., 2010; Gonzalez et al., 2018). In addition to its role in the regulation of GC responses, earlier reports have identified IL-21/IL-21R signaling as a switch factor for the secretion of all IgG subclasses, especially IgG_1_ and IgG_3_, as well as IgA by human splenic or peripheral naïve CD19^+^ B cells (Pene et al., 2004; Avery et al., 2008a). The B cell-intrinsic signaling via IL-21/IL-21R axis and the downstream STAT3 signaling have been accounted for the generation and the development of long-lived antibody responses (Avery et al., 2008b; Berglund et al., 2013). Consistent with these findings, mice lacking the IL-21R (*Il21r*^−/−^) failed to expand T cell-dependent, antigen-specific memory B cells and plasma cells (Rankin et al., 2011). Altogether, these observations demonstrate diverse biological functions for the IL-21/IL-21R axis in B cell-mediated immunity, spanning from the generation and the development of GCs to B cell proliferation, the antibody affinity maturation and the generation and the expansion of memory B cells.

**Figure 1:**
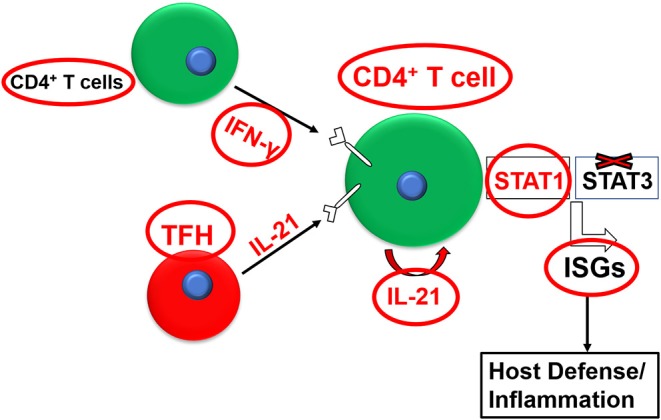
Synergy between the IL-21/IL-21R and the IFN-γ/IFNγR pathways for the optimal expression of interferon-stimulated genes (ISGs).

IL-21 can also regulate the effector function (e.g., cytokine production) of T cell subsets alone or in synergy with other cytokines (Strengell et al., 2002). IL-21 exhibits synergy with IL-15 for the enhancement of the T-bet expression and with IL-12 for induction of STAT4-dependent DNA binding in NK cells and T cells and the subsequent augmentation of T_H1_**-**polarized immune response, as evidenced by enhanced IFN-γ production (Strengell et al., 2002). IL-21 also showed synergy with IFN-γ to induce the optimal expression of a certain set of signature interferon-stimulated genes (ISGs) in humans or mice (Figure 1) (Wan et al., 2015; Solaymani-Mohammadi and Berzofsky, 2019). Interestingly, this effect on ISG expression was independent of STAT-3. These findings exemplify the breadth and the complexity of the IL-21/IL-21R signaling axis across species and in diverse biological settings (Strengell et al., 2002).

Since its discovery there has been an increasing body of evidence suggesting a variety of roles played by the IL-21/IL-21R signaling axis in the regulation of immunity and inflammation in several infectious and non-infectious settings (Ozaki et al., 2000; Sarra et al., 2010; Cupi et al., 2014; Wang et al., 2016). In recent years, the IL-21/IL-21R signaling axis has been implicated in the regulation of immunity and the pathogenesis in several bacterial, viral, fungal, and parasitic infections (Moretto and Khan, 2016; Solaymani-Mohammadi and Berzofsky, 2019; Spolski et al., 2019). The cytokine, or its cognate receptor, has been shown to be upregulated following infection with several protozoan and helminth parasites (Frohlich et al., 2007; Moretto et al., 2017). It has been suggested that the roles played by the IL-21/IL-21R signaling axis is context-dependent during microbial infections, including parasitic infections (Zhang Y. et al., 2016).

While the IL-21/IL-21R pathway has been shown to be important in protection against several microbial pathogens, the IL-21/IL-21R signaling axis has been linked to excessive inflammation and associated pathogenesis observed during infectious and non-infectious inflammatory conditions (Stolfi et al., 2011; Tortola et al., 2019). Consistent with this notion, dysregulated IL-21 expression is associated with multiple inflammatory conditions, including Crohn's disease, celiac disease and arthritis (Stolfi et al., 2011; Gensous et al., 2018; Wang et al., 2018). IL-21 is highly produced in human subjects with Crohn's disease and the blockade of the IL-21/IL-21R signaling axis in these patients resulted in reduced production of IFN-γ by mucosal lymphocytes (Monteleone et al., 2005). As such, the lack of an intact IL-21/IL-21R signaling axis in mice (*Il21r*^−/−^) resulted in attenuated inflammatory responses and reduced colitis in a murine model of human IBD (Wang et al., 2016). The activation of STAT3 in target cells has been proposed as a mechanism by which the IL-21/IL-21R signaling promotes tissue inflammation (Stolfi et al., 2011). These findings suggest that IL-21/IL-21R axis could be part of a positive feedback loop that amplifies an inflammatory response in the gut (Monteleone et al., 2005; Fina et al., 2008).

Despite the tissue distribution and the omnipresence of IL-21 target cells in tissues in which protozoan and helminth parasites reside, the role of the IL-21/IL-21R signaling pathway in protection against and/or the promotion of inflammation during parasitic infections is not fully understood. In recent years, however, it has been observed that IL-21 is induced in the periphery and mucosal tissues of humans and mice after infection with some parasites (Stumhofer et al., 2013; Carpio et al., 2015; Perez-Mazliah et al., 2015; Cai et al., 2016; Ryg-Cornejo et al., 2016; Moretto et al., 2017; Inoue et al., 2018; Wikenheiser et al., 2018) (Table 1). However, the exact mechanisms by which this signaling pathway regulates host immunity during parasitic infections are still unclear. Existing evidence suggests that the IL-21/IL-21R axis could play important roles in the development and the maintenance of anti-parasitic immunity and that targeting this signaling axis may provide an alternative approach for the development of new therapeutic agents for the prevention of parasite-induced immunopathology and tissue damage. Here, we review the roles of IL-21 and its receptor during protozoan and helminth infections in humans and in murine hosts.

**Table 1:** IL-21 in parasitic infections.

**Parasite Species**	**IL-21 Source/Cell type**	**References**
**Protozoa**		
*P. falciparum*	Plasma, serum; PBMCs*^a^* (CD4^+^ T cells)	Mewono et al., 2008, 2009; Moncunill et al., 2017; Oyegue-Liabagui et al., 2017
*P. vivax*	T_FH_ cells	Figueiredo et al., 2017
*P. chabaudi*	T_FH_ cells	Carpio et al., 2015; Perez-Mazliah et al., 2015
*P. berghei*	*γδ* T cells; T_FH_ cells	Ryg-Cornejo et al., 2016; Inoue et al., 2018
*P. yoelii*	T_FH_**-**like CD4^+^ T cells	Wikenheiser et al., 2018
*Babesia microti*	Plasma; CD4^+^CD44^+^ T cells	Djokic et al., 2018; Yi et al., 2018
*Toxoplasma gondii*	T_FH_ cells, Splenic CD4^+^ and CD8^+^ T cells; BMNCs*^b^* (CD4^+^ and CD8^+^ T cells)	Stumhofer et al., 2013; Moretto et al., 2017
*Trypanosoma cruzi*	Serum; T_H17_ cells	Cai et al., 2016; Natale et al., 2018
*Leishmania* spp.	IL-21 mRNA from skin lesions and spleen; T_FH_ cells, CD3^+^ T cells; dLNs^c^	Espitia et al., 2010; Ansari et al., 2011; Bollig et al., 2012; Gibson-Corley et al., 2012; Costa et al., 2015; Kong et al., 2017; Khatonier et al., 2018
**Helminths**		
Schistosomes	Plasma; LPLs*^d^*; T cells and non-T cells; T_FH_ cells; total splenocytes	Milner et al., 2010; Gringhuis et al., 2014; Wang et al., 2015; Torben et al., 2017
*Fasciola hepatica*	T_FH_ cells	Gringhuis et al., 2014
*Echinococcus granulosus*	Serum; PBMCs	Zhang et al., 2015
*Brugia malayi*	PBMCs	Babu et al., 2009; Anuradha et al., 2014
*Litomosoides sigmodontis*	TCL^e^; dLNs	Ritter et al., 2018
*Heligmosomoides polygyrus*	Intestinal mucosa; T_FH_ cells	Naradikian et al., 2016; Ariyaratne et al., 2018
*Aspiculuris tetrapetra*	Peyer's patches; spleen; mesenteric lymph nodes	Gomez-Samblas et al., 2018

a *Peripheral blood mononuclear cells*.

b *Brain mononuclear cells*.

c *Draining lymph nodes*.

d *Lamina propria lymphocytes*.

e *Thoracic cavity lavage*.

## Protozoan Parasites

### Blood-Dwelling Apicomplexa

#### *Plasmodium* spp.

IL-21 is induced during human infections with *Plasmodium falciparum* (Mewono et al., 2008, 2009) and *Plasmodium vivax* (Figueiredo et al., 2017), as well as following vaccination against *P. falciparum* (Moncunill et al., 2017). In one of the first reports on IL-21 induction in malaria, serum levels of IL-21, total parasite-specific IgG, and levels of IgG subclasses were measured in 73 Gabonese children positive for *P. falciparum* (Mewono et al., 2008). Positive correlations were observed between IL-21 plasma levels and *P. falciparum*-specific anti-erythrocyte-binding antigen-175 (EBA-175)-peptide 4, and IgG_1_ and IgG_3_ subclasses. EBA-175 peptide 4, a 42-mer peptide (1062–1103), is a vaccine candidate with a molecular weight of 175-kD that binds to a sialic acid-containing ligand expressed on the surface of erythrocytes and has been shown to be critical in parasite invasion of erythrocytes (Camus and Hadley, 1985). Recombinant antibodies generated against this protein inhibited the binding of merozoites to human RBCs *in vitro* (Sim, 1990; Sim et al., 1990). Interestingly, the study by Mewono et al. (2008) demonstrated that the plasma levels of IL-21 also positively correlated with the hemoglobin levels as well as the parasitemia, as a marker of the disease severity, in younger children with high parasite burdens. This study, however, did not investigate a potential correlation between the serum levels of IL-21 and the clinical phenotype.

In contrast to these findings, an independent clinical study performed to investigate the pro- and anti-inflammatory cytokine signature profiles in a distinct Gabonese population found no significantly elevated IL-21 levels in the sera of children infected with *P. falciparum* as compared with naive controls (Oyegue-Liabagui et al., 2017). This study demonstrated a reverse correlation between the serum IL-21 and the severity of anemia. Uninfected controls were enrolled from children with a febrile syndrome or a history of fever 48 h prior to the admission. Elevated levels of IL-21 in uninfected controls could be due to non-*P. falciparum* induction of IL-21 production and may, in part, explain why no significant differences were observed between the levels of IL-21 in the sera of *P. falciparum*-infected human subjects as compared with uninfected controls. As such, the lack of significant elevation in plasma IL-21 between *P. falciparum*-infected as compared with uninfected control groups could be partially explained by the inclusion of uninfected controls concurrently infected with other parasitic or non-parasitic pathogens commonly found in areas where human *P. falciparum* infection is endemic (Dejon-Agobe et al., 2018; M'Bondoukwe et al., 2018). These two clinical studies did not measure levels of the *P. falciparum*-induced IL-21 production in human subjects with *P. falciparum* infections as well as in uninfected controls (Mewono et al., 2008; Oyegue-Liabagui et al., 2017). This lack of IL-21 production specificity may render some of the results of these two studies inconclusive.

A follow-up study by Mewono et al. (2009) demonstrated that IL-21 was induced following the stimulation of peripheral blood mononuclear cells (PBMCs) isolated from *P. falciparum*-experienced human subjects as compared with *P. falciparum*-naïve controls in response to two malaria vaccine candidates, the *P. falciparum* glutamate-rich protein (PfGLURP) or the *P. falciparum* merozoite surface protein 3 (PfMSP-3) (Mewono et al., 2009). Interestingly, PBMCs isolated from the venous blood of both *P. falciparum*-experienced and *P. falciparum*-naïve controls used in a recall assay secreted IL-21 in response to either PfGLURP or PfMSP-3, with human subjects exposed to *P. falciparum* producing significantly higher levels of *P. falciparum* peptide-induced IL-21 (Mewono et al., 2009).

PfGLURP or PfMSP-3 are expressed by all developmental stages of *P. falciparum* (pre-erythrocytic and erythrocytic stages) and on the surface of *P. falciparum* merozoites, respectively (Jordan et al., 2009; Kaur et al., 2017). These two *P. falciparum*-derived proteins are considered highly immunogenic and have been considered as vaccine candidates alone or in combination as fused proteins (e.g., GMZ2, a fusion of PfGLURP and PfMSP3) in clinical settings (Oeuvray et al., 1994a,b; Roussilhon et al., 2007; Belard et al., 2011). The PfGLURP- or PfMSP-3-elicited IL-21 production by PBMCs derived from *P. falciparum*-experienced human subjects may suggest potential roles for this cytokine to mediate immunity to *P. falciparum* infections and may further provide a surrogate biomarker to measure the efficacy of malaria vaccines. These finding were further augmented by the observations that RTS, S/AS01E, a vaccine consisting of sequences of the *P. falciparum* circumsporozoite (CS) protein (RTS,S) and the hepatitis B surface antigen (HBsAg) induced the intracellular expression of IL-21 by antigen-specific CD4^+^ T cells in pediatric subjects enrolled in a phase III clinical trial before and 1 month after initial vaccination (Moncunill et al., 2017).

IL-21 expression during human malaria has been mostly studied in the context of malaria infections caused by *P. falciparum* (Moncunill et al., 2017). However, further clinical studies demonstrated that uncomplicated *P. vivax* malaria infection in a Brazilian population induced significantly higher levels of serum IL-21 production and also promoted the expansion of T_FH_ cells, defined as PD-1^+^ICOS^+^CXCR5^+^CD45RO^+^CD3^+^CD4^+^ cells, in these patients (Figueiredo et al., 2017). This study also showed the propensity of *P. vivax*-induced T_FH_ cells to secret IL-21 and to induce immunoglobulin production (IgG_1_ and IgG_3_) by B cells upon being co-cultured with naïve B cells (Figueiredo et al., 2017).

IL-21 is expressed in several murine malaria models, including *P. berghei* (Ryg-Cornejo et al., 2016; Inoue et al., 2018), *Plasmodium chabaudi* (Perez-Mazliah et al., 2015; Sebina et al., 2017) and *Plasmodium yoelii* (Wikenheiser et al., 2018). Genetic disruption of the IL-21/IL-21R singling axis compromised *P. chabaudi*-specific IgG responses and resulted in the impairment of memory B cell generation. The IL-21/IL-21R axis disruption also led to impaired resolution of parasitemia in the chronic phase of infection (Perez-Mazliah et al., 2015). It also has been suggested that IL-21 promotes the humoral immune response against *P. chabaudi chabaudi*, as evidenced by the lack of the extrafollicular plasmablast development within the first week following the infection (Sebina et al., 2017). Mice deficient in either IL-21 (*Il21*^−/−^) or IL-21R (*Il21r*^−/−^) were susceptible to rechallenge infections with homologous murine malaria parasites (Perez-Mazliah et al., 2015). Studies using the mixed bone marrow chimeric mice also indicated that T-cell-derived (i.e., T_FH_ cells) IL-21 interacted with the IL-21R expressed on B cells for the effective control of parasitemia during *P. chabaudi* infection, highlighting the requirement of T-cell/B cell crosstalk for the development and the maintenance of a robust and protective anti-malaria immune response in the mouse (Perez-Mazliah et al., 2015).

Interestingly, IL-21 and IFN-γ were co-expressed by T_FH_ cells generated during the erythrocytic phase of *P. chabaudi* infection in mice (Perez-Mazliah et al., 2015). The IFN-γ-expressing T effector cells generated following *P*. *chabaudi* were predominantly positive for CXCR5, a marker for T_FH_ cells, and acquired the ability to express IL-21 and IFN-γ, later in the course of infection and in the memory phase of the immune response (Carpio et al., 2015). Generally, the regulation of the classical CXCR5^hi^PD-1^hi^ GC T_FH_ cell subset requires Bcl6, a master transcription factor critical for the differentiation of T_FH_ cells. However, the regulation of the CXCR5^+^IL-21^+^IFN-γ^+^ subset was independent of Bcl6 during infection of *P*. *chabaudi* in the mouse (Carpio et al., 2015).

Murine *P. berghei* ANKA infection as well as immunization with irradiated *P. berghei* ANKA (iPbA) induced IL-21-expressing T_FH_ cells (Ryg-Cornejo et al., 2016). CXCR5^int^PD-1^int^ T_FH_ cells were the primary source of IL-21 expression early during the course of infection, whereas T_FH_ cells with high expression of CXCR5 and PD-1 (CXCR5^high^PD-1^high^) also expressed IL-21 during the resolution phase of the infection (Ryg-Cornejo et al., 2016). Interestingly, infection with *P. berghei*, but not vaccination with iPbA, could remodel the microstructure of the splenic GC and results in impaired T_FH_ cells differentiation (Ryg-Cornejo et al., 2016).

In addition to T_FH_ cells, other immune cell types, including γδ T-cells and a T_FH_**-**like CD4^+^ T cell population expressing the surface molecule, NK1.1, and T_FH_ cell markers simultaneously, have also been shown to express IL-21, as well as IFN-γ, during the early phase of infection with *P. berghei* and *P. yoelii*, respectively (Inoue et al., 2018; Wikenheiser et al., 2018). The importance of the IL-21 expression by non-conventional T_FH_ cells was reinforced by the observations that mice lacking γδ T-cells were incompetent to control *P. berghei* infection and that the lack of ability to control infection correlated with the findings that γδ T-cell-deficient mice had impaired levels of *P. berghei*-specific antibodies, decreased numbers of T_FH_ cells and fewer GC B cell toward the later course of infection as compared with their C57BL/6 counterparts (Inoue et al., 2018). Blockade of the IL-21R using the IL-21R-Fc led to a reduction in T_FH_ cells and GC B cell numbers toward the later phase of infection, suggesting the importance of the IL-21 expressed by γδ T-cell during the early phase of infection to promote the development of T_FH_ cells as well as the GC B cells during the later course of infection (Inoue et al., 2018). Altogether, these findings exemplify the requirement of the IL-21/IL-21R signaling axis for development of the GC reaction, the promotion of high-affinity parasite-specific antibody repertoire generation by B cells, and the regulation of the antibody class switching in these cells during experimental murine malaria infections (Perez-Mazliah et al., 2015).

#### *Babesia* sp.

The evidence supporting potential protective roles of the IL-21/IL-21R signaling axis during experimental murine babesiosis is limited (Djokic et al., 2018; Yi et al., 2018). No significant differences were observed in the plasma levels of IL-5, IL-17A, and IL-21 in *Babesia microti*-infected C3H/HeJ mice as compared with uninfected controls (Djokic et al., 2018). A T_H1_**-**mediated immunity, as opposed to a T_H17_**-**biased immune response, was critical for the clearance of *B. microti* in susceptible C3H/HeJ mice (Djokic et al., 2018). Conversely, *B. microti* was shown to elicit a strong immune response in C57BL/6 mice characterized by the induction of a GC reaction accompanied by the promotion of T_FH_ cells generation and increased levels of the T cell effector cytokines, including IL-21 and IFN-γ (Yi et al., 2018). This study also showed that T_FH_ cells expanded upon *B. microti* infection. However, the specific expression of IL-21 by T_FH_ cells, as one of the major expressors of IL-21, was not determined following *B. microti* infection in the mouse (Yi et al., 2018). The differences in host strain susceptibility to *B. microti* infection, age discrepancies and the dose of inoculum could account for inconsistent findings observed between these two studies. Collectively, the lack of experimental evidence demonstrating a direct and protective role played by the IL-21/IL-21R signaling axis precludes a definite conclusion as to how this signaling axis regulates host immunity during experimental babesiosis and may warrant further future studies.

### Tissue-Dwelling Apicomplexa

#### Toxoplasma gondii

It has been suggested that experimental toxoplasmosis elicits a strong T_FH_ cell response and that IL-21 plays important roles in the regulation of T cell responses in an encephalitis susceptible mouse model of the disease (Moretto et al., 2017). Nevertheless, the survival rate of *Il21r*^−/−^ mice infected with *T. gondii* was comparable with their wild-type counterparts, despite the significant impairment of IgG_1_ in the serum of *Il21r*^−/−^ mice (Ozaki et al., 2002).

To further dissect the roles of IL-21 in defining humoral immunity and its roles in the maintenance of a balance between protective and pathogenic immune response during acute *T. gondii* infection, it was demonstrated that a larger proportion of IL-21-expressing CD4^+^ and CD8^+^ T cells were found in the brain of wild-type mice chronically infected with *T. gondii* (Stumhofer et al., 2013). As such, the requirement of IL-21 for the control of the *T. gondii* chronic infection was also confirmed by increased parasite burdens in the brains of chronically infected *Il21*^−/−^ mice as compared with their wild-type counterparts (Stumhofer et al., 2013). Interestingly, *Il21*^−/−^ mice showed no immune-mediated pathology during the acute phase of infection and IgG production was impaired in these mice as compared with wild-type controls and the impairment in IgG production in *Il21*^−/−^ mice correlated with diminished numbers of GC B cells (Stumhofer et al., 2013).

It has previously been shown that IL-21 acts synergistically with several cytokines, including IL-2 (Battaglia et al., 2013), IL-15 (Kishida et al., 2003; Strengell et al., 2003; Zeng et al., 2005; Li et al., 2014), and IL-18 (Lusty et al., 2017) to exert some of its biological functions. To explore the efficacy of a DNA vaccine (pVAX/TgMIC8) encoding the *T. gondii* microneme protein 8 (MIC8), mice were immunized in the absence or the presence of eukaryotic plasmids expressing murine IL-15 (pVAX/mIL-15), IL-21 (pVAX/mIL-21), or both (pVAX/mIL-21/mIL-15). Mice immunized with pVAX/TgMIC8 alone or combined with pVAX/mIL-15 and, to a lesser extent IL-21 (pVAX/mIL-21), mounted strong humoral T_H1_ immune profiles (Li et al., 2014). Interestingly, mice co-administered with a combination of pVAX/TgMIC8 and plasmids expressing both murine IL-15 and IL-21 (pVAX/mIL-21/mIL-15) elicited the strongest immune profiles as compared with controls, leading to a significant increase in survival and decreased numbers of tissue cysts in the brain (Li et al., 2014). Similarly, vaccination with the *T. gondii* calcium-dependent protein kinase 1 (TgCDPK1) combined with plasmids expressing both murine IL-15 and IL-21 (pVAX/mIL-21/mIL-15) significantly increased survival time and led to reduced cyst numbers in the brain of mice receiving a combined pVAX-CDPK1/pVAX/mIL-21/mIL-15 vaccine regimen as compared with those mice receiving either pVAX-CDPK1 or pVAX/mIL-21/mIL-15 alone (Chen et al., 2014). These findings may demonstrate a synergy between IL-21 and IL-15 for the optimal expression of parasite-specific humoral and cellular immune responses following vaccine administration. IL-21, in collaboration with IFN-γ, has also been found to be required for the optimal expression of several interferon-stimulated genes, including multiple MHC class molecules and chemokine/chemokine receptors (Solaymani-Mohammadi and Berzofsky, 2019). Altogether, these observations further suggest that the inclusion of IL-21, in combination with other cytokines, as an adjuvant may lead to the optimal expression of molecules required for T cell-antigen-presenting cell (APC) interactions and may enhance the efficacy of the vaccines against *T. gondii* infection.

### Blood-Dwelling Kinetoplastids

#### Trypanosoma cruzi

T_H17_-mediated immune responses have been mostly studied in the context of extracellular bacterial and fungal infections (Curtis and Way, 2009; Zelante et al., 2009; Sellge et al., 2010; Das and Khader, 2017). However, it has been demonstrated that *T. cruzi* infection elicits a strong proinflammatory cytokine profile characterized by increased expression of IFN-γ, TNF-α, and IL-17 (Silva et al., 1995; Michailowsky et al., 2001; Miyazaki et al., 2010). The T_H17_ response is necessary for acute resistance to *T. cruzi* and the transcription factor T-bet regulates *T. cruzi*-specific T_H17_ responses (Guo et al., 2009). Consistent with the protective roles of T_H17_ during *T. cruzi* infection, T_H17_ cells provide help to CD8^+^ T cells in an IL-21-dependent manner (Cai et al., 2016). Interestingly, *Rag*^−/−^ mice reconstituted with *Il21r*^−/−^ CD8^+^ T cells had consistently higher parasitemia levels as compared with those *Rag*^−/−^ mice receiving wild-type CD8^+^ T cells and mice in the former group died before day 20 p.i. (Cai et al., 2016). These findings suggested a protective role for IL-21 in the T_H17−_mediated resistance via providing help to CD8^+^ T cells during experimental *T. cruzi* infection in murine hosts.

The serum levels of several cytokines, including IL-21, positively correlated with disease severity in patients with chronic Chagas disease caused by *T. cruzi* (Natale et al., 2018). Consistently, lower levels of IL-21 production were observed in the sera of human subjects with less severe cardiac dysfunction as compared with those presented with an advanced cardiac dysfunction profile (Natale et al., 2018). These observations may suggest a potential role for the IL-21/IL-21R axis in the promotion of pathology in chronically-infected human subjects. However, the mechanisms by which the IL-21/IL-21R signaling pathway may mediate the pathogenesis of acute and chronic *T. cruzi* infections still remain unclear and may warrant further investigations.

### Tissue-Dwelling Kinetoplastids

#### *Leishmania* spp.

IL-21 transcripts are detectable following cutaneous leishmaniasis caused by *Leishmania panamensis, Leishmania major*, and *Leishmania braziliensis* infections as well as in humans and mice infected with *Leishmania donovani*, the causative agent of visceral leishmaniasis (Espitia et al., 2010; Bollig et al., 2012; Costa et al., 2015; Kong et al., 2017; Khatonier et al., 2018). IL-21 expression significantly and positively correlated with expression of IFN-γ and IL-27, but not FoxP3 or TGF-β, in human subjects with *L*. *braziliensis* cutaneous lesions (Costa et al., 2015). CD3^+^ T cells were the major source of IL-21 expression during visceral leishmaniasis in humans (Ansari et al., 2011). Further, recombinant human IL-21 could augment antigen-specific IL-10 production in a whole-blood assay (Ansari et al., 2011). However, an earlier report failed to establish a connection between the lack of IL-21R signaling and impaired T_H1_**-**biased immunity against *L. major* infection (Frohlich et al., 2007). In this study, *Il21r*^−/−^ mice mounted a strong T_H1_ immune response against *L. major* infection as evidenced by a higher percentage of antigen-specific IFN-γ**-**producing CD4^+^ T cells and these mice had comparable footpad swellings as compared with C57BL/6 controls (Frohlich et al., 2007). These findings suggest that the IL-21/IL-21R signaling axis was not required for host defense against *L. major* and that the absence of IL-21R signaling was not critical for the development of a T_H1_**-**biased response against this intracellular protozoan parasite.

Host strain-specific susceptibility or resistant to infection is considered an important determinant of disease outcome in parasitic diseases (Araujo et al., 1976; Ruebush and Hanson, 1979; Ishii and Sano, 1989; Duleu et al., 2004). Varied susceptibility of C57BL/6 (resistant) and Balb/c (susceptible) strains to *L. major*, for example, represents a classical dichotomy as to how host's genetic background can contribute to disease outcome following leishmanial infections (Lohoff et al., 1989; Scott, 1991; Sommer et al., 1998; Barbi et al., 2008). Several mechanisms have been proposed as to why some mouse strains are more susceptible to a given parasite while others do not (von Stebut et al., 1998; Rosas et al., 2005; Nigg et al., 2007). These may include discrepancies in the magnitude of a particular immune pathway and/or the differential expression of immune compartments and/or immune effector molecules between mouse strains (von Stebut et al., 2000; Von Stebut et al., 2003; Hurdayal et al., 2013; Sassi et al., 2015). Analysis of the GC B cell response in the draining lymph nodes (dLNs) of C3HeB/FeJ and C57BL/6 mice (which develop healing and non-healing lesions, respectively) in a *L. major*/*Leishmania amazonensis* model of co-infection revealed that the inability of C57BL/6 mice to resolve leishmanial lesions correlated with reduced numbers of GC B cells and memory B cells and fewer parasite-specific antibody-producing cells as compared with the C3HeB/FeJ strain (Gibson-Corley et al., 2012). In this study, the differences in the local production of IL-21 or different expression levels of IL-21R did not explain the distinct clinical profiles observed between the two strains, since the two strains had comparable local production and expression of IL-21 and IL-21R, respectively (Gibson-Corley et al., 2012). These findings may suggest that varied clinical profiles in the mouse strains developing healing (C3HeB/FeJ) vs. non-healing (C57BL/6) lesions can be mainly due to the mouse strain-dependent discrepancies in intrinsic T_FH_ cell or B cell functional properties.

It is well-established that the IFN-regulatory factor (IRF) family of transcription factors are key players in T_H_ differentiation; whereas IRF1 is crucial for T_H1_ differentiation (Lohoff et al., 1997; Lohoff and Mak, 2005), IRF4 is required for T_H2_ and T_H17_ cell differentiation (Brustle et al., 2007; Huber et al., 2008). Mice deficient for IRF4 (*Irf4*^−/−^) were characterized by their failure to form GCs in the draining popliteal lymph nodes in response to chronic *L. major* infection as well as their inability of dLN**-**derived T_FH_ cells to optimally express and secrete IL-21 at both the mRNA and protein levels, respectively (Bollig et al., 2012). These observations may suggest an important role played by IRF4 in T_FH_ cells**-**derived IL-21 expression in response to a chronic, intracellular parasitic pathogen.

## Helminth Parasites

### Trematodes

#### Schistosomes

Several studies have demonstrated that IL-21 is induced at both the mRNA and protein levels during natural infections with several species of schistosomes in humans and in murine models of schistosomiasis (Milner et al., 2010; Bourke et al., 2013; Zhang Y. et al., 2016), as well as following vaccination with schistosome-derived peptides (Zhang et al., 2013, 2018; Bourke et al., 2014; Fairfax et al., 2015). Ileal lamina propria lymphocytes-(LPLs) and axillary lymph node-derived T cells and non-T cell subsets from uninfected rhesus macaques (*Macaca mulatta*) secreted high levels of IL-21 in a recall assay in response to *S. mansoni* (Sm) egg antigen (SmEA) and worm antigen (SmWA) preparations *ex vivo* (Torben et al., 2017). Dendritic cell-specific adhesion molecule-3-grabbing non-integrin (DC-SIGN, CD209) controls the differentiation of T_FH_ cells via IL-27 expression during infections with *S. mansoni* and the liver fluke, *Fasciola hepatica* (Gringhuis et al., 2014). Interestingly, the transcription factor, IFN-stimulated gene factor 3 (ISGF3) induces the expression of the IL-27 subunit p28 by primed DCs, and IL-27 produced by DCs is required for the differentiation of T_FH_ cells and the subsequent IL-21 expression by T_FH_ cells during infections with trematodes, including *S. mansoni* and *F. hepatica* (Gringhuis et al., 2014).

In contrast to potential roles played by the IL-21/IL-21R signaling axis in protective immunity during infection with trematodes, earlier studies demonstrated that the IL-21/IL-21R signaling pathway promotes the induction of tissue damage following *Schistosoma* spp. infections (Pesce et al., 2006). While parasite burdens were comparable between wild-type mice and mice lacking a functional IL-21/IL-21R signaling axis (*Il21r*^−/−^), the lack of a functional IL-21/IL-21R signaling pathway in *Il21r*^−/−^ mice infected with *S. mansoni* led to diminished T_H2_-dependent immunopathology in those mice, as evidenced by attenuated formation of granulomatous inflammation and liver fibrosis (Pesce et al., 2006). Mechanistically, the impaired *S. mansoni*-induced granulomatous formation in *Il21r*^−/−^ mice correlated with a significant impairment in both the tissue expression and the function of T_H2_ cytokines, including IL-4 and IL-13, required for the fibrotic tissue responses during *S. mansoni* infection (Pesce et al., 2006). Furthermore, IL-21 treatment of the bone marrow**-**derived macrophages (BMDM) promotes the expression of cognate receptors for IL-4 and IL-13 (IL-4Rα and IL-13Rα1) in macrophages, indicating that the augmentation of alternate macrophage activation can occur in an IL-21/IL-21R axis**-**dependent manner (Pesce et al., 2006).

It is known that the ICOS/ICOSL axis contributes to the T cell differentiation, T cell activation and its optimal functions (Hutloff et al., 1999; Dong et al., 2001). Mice deficient in either a functional ICOS or ICOSL (*Icos*^−/−^ and *Icosl*^−/−^ mice, respectively) had diminished levels of serum antibodies, defective antibody class switching and impaired GC formation and T_H17_ cell development (Dong et al., 2001; Nurieva et al., 2003). Consistent with the requirement of the ICOS/ICOSL signaling pathway in the development and the function of T_H17_ cells, *Icosl*^−/−^ mice infected with *Schistosoma japonicum* showed decreased expression of T_H17_ cytokines, including IL-21 (Wang et al., 2015). The impaired T_H17_ cytokine expression in *Icosl*^−/−^ mice correlated with attenuated liver granulomatous inflammation around parasite eggs and the significant reduction in hepatic fibrosis in those mice as compared with wild-type controls (Wang et al., 2015).

Interestingly, IL-21 does not directly promote the production of the *Schistosoma* egg-induced IL-17 production by antigen-specific CD4^+^ T cells. Antibody neutralization of IL-21 in a DC-CD4^+^ T cells co-culture setting, in which DCs are pulsed with egg antigens and co-cultured with naïve CD4^+^ T cells, did not impair the antigen-specific IL-17 production by antigen-specific CD4^+^ T cells (Shainheit et al., 2008). This is consistent with studies demonstrating that the lack of the IL-21/IL-21R signaling axis does not impair the secretion of IL-17, as well as IFN-γ, following colonic inflammation (Wang et al., 2016; Solaymani-Mohammadi and Berzofsky, 2019). It is likely that the IL-21 promotion of tissue inflammation and immunopathology during schistosomiasis occurs via other signaling pathways independent of the IL-17/STAT3 signaling axis, most likely through STAT1 or STAT5 signaling.

The increased levels of IL-21 in human subjects with schistosomiasis as compared with healthy controls (Milner et al., 2010) and the fact that mice deficient for the IL-21/IL-21R signaling pathway have significantly reduced parasite-induced tissue fibrosis suggest that this axis could play important roles in parasite-induced pathogenesis. Targeting this signaling axis may provide an alternative approach for the development of new therapeutic targets for the prevention of fibrotic reactions that develop during human infections with different species of *Schistosoma*.

### Cestodes

#### Cystic Echinococcosis Caused by *Echinococcus granulosus*

In a clinical study, the roles played by T_FH_ cells and the cytokines they secrete, including IL-4 and IL-21, during different stages of human cystic echinococcosis (CE) were investigated in patients with active, transitional or inactive disease (Zhang et al., 2015). The findings of this study demonstrated that the percentage of circulating T_FH_ cells, defined as CCR7^lo^PD-1^hi^CXCR5^+^CD4^+^, increased in the PBMCs of patients with active and transitional disease as did the serum levels of IL-21 and IL-4 in those patients as compared with healthy controls (Zhang et al., 2015). The expression of IL-21 and IL-4 by PMBCs isolated from patients with CE significantly increased in response to the hydatid fluid as compared with PMBCs from healthy volunteers (Zhang et al., 2015). Interestingly, CE patients with inactive disease neither had increased percentages of circulating T_FH_ cells nor did they show increased levels of IL-21 and IL-4 as compared with healthy controls. These observations suggest that T_FH_ cells may be involved in host immunity or immunopathology associated with CE in humans via the expression of canonical cytokines expressed by these cells. However, the causality, the direction of any causality and the potential mechanisms underlying the differences in the T_FH_ cells and cytokine profiles between human subjects with active, transitional or inactive CE still require further investigation.

### Nematodes

#### Tissue- and Lumen-Dwelling Nematodes

Limited evidence suggests that T_H17_ cytokines, including IL-17A, IL-17F, IL-21, and IL-23, are elevated in human subjects with lymphatic filariasis as well as in animal models of human filariasis and this cytokine signature is associated with helminth-induced immunopathology in chronic lymphatic filariasis (Babu et al., 2009; Anuradha et al., 2014; Ritter et al., 2018).

The roles played by the IL-21/IL-21R signaling axis in the maintenance of intestinal immune homeostasis in the context of intestinal helminthiasis is not fully-understood. IL-21 was detectable following infection of mice with a natural murine intestinal nematode, *Heligmosomoides polygyrus* (Espitia et al., 2010; Ariyaratne et al., 2018). The expression of IL-21 in another murine model of intestinal nematodiasis, caused by the mouse pinworm, *Aspiculuris tetraptera*, was tissue-dependent and significantly higher levels of IL-21 transcripts were measured in the Peyer's patches (PP), and to a lesser extent in the spleens and in the mesenteric lymph nodes, of wild-type mice infected with *A. tetraptera* (Gomez-Samblas et al., 2018). Mice deficient for a functional IL-21/IL-21R axis failed to elicit anti-helminth-specific IgG_1_ responses and subsequently were unable to clear helminth infection as compared with their wild-type counterparts (King et al., 2010). The IL-21/IL-21R axis was important for driving the differentiation of *H. polygyrus*-specific CD138^+^ plasma cells following intestinal infection (King et al., 2010). Despite the observations that *Il21r*α^−/−^ had impaired differentiation of CD138^+^ plasma cells 2 weeks post-infection, this study did not find evidence demonstrating any roles for IL-21R signaling in GC formation, isotype class switching or T_H2_ differentiation (King et al., 2010). In contrast, it appears that the IL-21/IL-21R signaling axis also played critical roles in the development of T_H2_ immune response following infection with the intestinal luminal nematodes, *Nippostrongylus brasiliensis* and *H. polygyrus* (Frohlich et al., 2007). Mice deficient for the IL-21R (*Il21r*^−/−^) showed reduced accumulation of immune subsets in the intestine, including lymphocytes, and reduced sizes and numbers of intestinal granuloma following helminth infections, suggesting a critical requirement of the IL-21/IL-21R axis for mounting T_H2_ responses (Frohlich et al., 2007). These findings may also suggest a role for this signaling pathway in the initiation and the promotion of inflammation and tissue immunopathology during intestinal inflammatory condition, as observed in other models of colonic infections (Solaymani-Mohammadi and Berzofsky, 2019).

In another line of research, it was found that both IL-21 and IFN-γ can drive the expression of T-bet transcripts (*Tbx21*) and the intranuclear expression of T-bet protein in follicular B cells in the presence of TLR7 or TLR9 agonists *in vitro* (Naradikian et al., 2016). This study also demonstrated that the IFN-γ-induced expression of T-bet was independent of either IL-4 or IL-21, since IFN-γ alone was sufficient for driving the expression of T-bet in follicular B cells (Naradikian et al., 2016). In contrast, the stimulation of follicular B cells with IL-21 led to the expression of CD11c, whereas IFN-γ did not alter CD11c expression by those cells. Considering that the IL-21/IL-21R signaling axis signals mainly via STAT3, but also STAT-1 and STAT5, it is likely that the expression of CD11c is mediated by the IL-21-induced activation of STAT3 or STAT5, whereas the IL-21- or IFN-γ-induced expression of T-bet may occur mainly via the STAT1 pathway, a pathway via which both cytokines exert parts or all of their biological functions. Studies in a T_H2_-biased murine model of infection with *H. polygyrus* further confirmed that the two main cytokines secreted by T_FH_ cells, IL-21 and IL-4, can cross-regulate the expression of T-bet and CD11c in the absence of an intact IFN-γ/IFN-γR signaling axis (Naradikian et al., 2016).

## Conclusion

To conclude, we and others already have shown that, in synergy with IFN-γ, the IL-21/IL-21R axis is required for the optimal expression of ISGs, including several chemokines and chemokine receptors, as well MHC class molecules required for protection against parasitic infections, in a STAT3-independent, most likely STAT1-dependent, manner in humans or in mice (Wan et al., 2015; Solaymani-Mohammadi and Berzofsky, 2019). Based on this, the use of recombinant human IL-21 in vaccine regimens in clinical trials (for example targeting *P. falciparum* infections) may provide novel and unique vaccine strategies to elicit an effective anti-malarial immune response and warranting a better protective outcome. Most studies investigating the roles played by the IL-21/IL-21R axis in parasitic infections, whether protective or detrimental, stemmed from reports in animal models. However, studies in animal models require careful interpretation and cannot be simply extrapolated to humans since animal immune responses, including murine hosts, are not always equivalent to those observed in humans. For instance, studies have utilized *P. berghei*, a rodent malaria parasite, to analyze the extent to which the IL-21/IL-21R axis contributes to host immunity during CM in the murine hosts as a surrogate for the human disease (Inoue et al., 2018). Multiple studies, however, have highlighted significant differences as to how *P. berghei* induces CM in mice and that the murine infection with *P. berghei* does not recapitulate all aspects of the human disease (reviewed in Riley et al., 2010; Stevenson et al., 2010). Unveiling the IL-21/IL-21R crosstalk with other cytokine networks and their downstream genes will provide insight into the development of novel therapeutic targets for the parasite-induced immunopathology and the control of parasitic infections.

## Author Contributions

SS-M conceived the study, contributed hypotheses, and wrote the manuscript. LE and SS critically read the manuscript and provided feedback.

### Conflict of Interest

The authors declare that the research was conducted in the absence of any commercial or financial relationships that could be construed as a potential conflict of interest.
